# Glucose Metabolism in Acute Myeloid Leukemia Cell Line Is Regulated via Combinational PI3K/AKT/mTOR Pathway Inhibitors

**DOI:** 10.5812/ijpr-140507

**Published:** 2023-11-14

**Authors:** Abbas Ranjbar, Mohsen Soltanshahi, Saeid Taghiloo, Hossein Asgarian-Omran

**Affiliations:** 1 Department of Immunology, School of Medicine, Mazandaran University of Medical Sciences, Sari, Iran; 2 Student Research Committee, Mazandaran University of Medical Sciences, Sari, Iran; 3 Gastrointestinal Cancer Research Center, Non-Communicable Diseases Institute, Mazandaran University of Medical Sciences, Sari, Iran

**Keywords:** PI3K/AKT/mTOR, Glycolysis, Citric Acid Cycle, Acute Myeloid Leukemia, Signaling Pathways

## Abstract

**Background:**

Metabolism reprogramming is a survival mechanism in acute myeloid leukemia (AML) cells in the tumor microenvironment. Therefore, we investigated the effect of signaling pathway inhibitors on the expression of genes rewired in the metabolic pathway of AML cells.

**Methods:**

HL-60 cells were treated with Idelalisib, MK-2206, and Everolimus, which respectively are selective inhibitors of phosphatidylinositol-3-kinase (PI3K), AKT, and the mammalian target of rapamycin (mTOR), either individually or in combination. The relative expressions of glucose transporter 1, hexokinase 2, pyruvate kinase, pyruvate dehydrogenase E1, citrate synthase, isocitrate dehydrogenase 2, and hypoxia inducible factor 1 subunit alpha were determined by real-time PCR.

**Results:**

The combined treatment of HL-60 cells with Idelalisib, MK-2206, and Everolimus decreased the expression of glucose transporter 1, hexokinase 2, pyruvate kinase M2, pyruvate dehydrogenase E1, citrate synthase, isocitrate dehydrogenase 2, and hypoxia inducible factor 1 subunit alpha.

**Conclusions:**

A combination of PI3K/AKT/mTOR pathway inhibitors regulates the expression of genes involved in glycolysis, pyruvate dehydrogenase complex (PDH), and the tricarboxylic acid (TCA) cycle and interferes with metabolic reprogramming and immune evasion mechanisms of AML leukemic cells. Combinational therapy approaches to block these pathways might be a promising and novel therapeutic strategy for targeting the metabolic requirements of AML cells.

## 1. Background

Acute myeloid leukemia (AML) is a hematopoietic malignancy characterized by heterogeneous molecular and clinical outcomes (1). Considering conventional chemotherapy and hematopoietic stem cell transplant, the 5-year overall survival for patients less than and over 60 years is about 40% and 10 - 20%, respectively (2). However, more than 50% of AML patients will relapse after complete remission through chemotherapy, and 20% will show primary refractory disease (2). Therefore, new treatment strategies were developed to treat AML due to the high toxicity of chemotherapeutic agents and resistance to standard therapies (3). Meanwhile, small-molecule inhibitors that target signaling pathways are a promising therapeutic approach (3).

Moreover, aberrantly upregulated intracellular signaling pathways contributed to cell cycle progression, uncontrolled proliferation, and resistance to chemotherapy in tumor cells (4). Oncogenic activation of phosphatidylinositol-3-kinase (PI3K), AK strain transforming (AKT), and mechanistic/mammalian target of rapamycin (mTOR) pathways are the most frequently activated in cancers, including AML patients (5). Therefore, several small molecules developed during the last two decades have inhibited the PI3K/AKT/mTOR pathway selectively (6). The efficacy of these drugs has been evaluated through preclinical and clinical trials. In this regard, Idelalisib, MK-2206, and Everolimus as selective inhibitors of PI3K, AKT, and mTOR, respectively, are currently in different studies as monotherapy or in combination with other conventional treatments (5, 7). Recent data indicated that combinational therapy with these drugs was more effective than single inhibition of these pathways in AML (8).

On the other hand, alterations in cell metabolism and metabolic rewiring are distinctive features of many cancers, including AML, providing survival, growth, and adaptation to the microenvironment (9). Furthermore, changing the metabolic pathways in cancerous cells led to chemotherapy resistance and immune evasion of tumor cells (10). Because of the high proliferation rate, shifting metabolic pathways is essential for tumor cells to supply anabolic needs, including energy and macromolecules like nucleic acids, lipids, and proteins (11). Aerobic glycolysis is one of the crucial pathways that tumors preferentially use to metabolize most of the glucose, even in the presence of oxygen (12). Like aerobic glycolysis, evidence has highlighted that the atypical expression of several crucial enzymes in the tricarboxylic acid (TCA) cycle contributes to the conditions required for anabolic processes. Also, the gene mutation in the TCA cycle resulted in the accumulation of oncometabolites that shed light on epigenetic landmarks (13).

## 2. Objectives

Thus, the investigation of the molecular basis of immunological-metabolic crosstalk has become a prime purpose. To elucidate the effect of signaling pathways on the expression of genes involved in the metabolic activity of AML cells, we investigated the relationship between the inhibitors of the PI3K/AKT/mTOR pathway and the expression of genes in glycolysis, the pyruvate dehydrogenase (PDH) complex, and the TCA cycle in AML cells. It was also looked at whether the immune checkpoint ligands and the expression of these genes were correlated following treatment with PI3K/AKT/mTOR inhibitors.

## 3. Methods

### 3.1. Reagents

The drugs Idelalisib, MK-2206, and Everolimus were supplied by the Cayman Chemical Company (Michigan, United States) as inhibitors of PI3K, AKT, and mTOR, respectively. They were dissolved in dimethyl sulfoxide (DMSO) and kept frozen in aliquots.

### 3.2. Cell Culture

The AML cell line HL-60 was obtained from the Pasteur Institute of Iran (Tehran, Iran). Cells were cultured in RPMI-1640 medium (Biowest, Nuaille, France) supplemented with 10% FBS (Biowest, Nuaille, France), 100 U/mL penicillin G, and 100 µg/mL streptomycin, and maintained at an atmosphere of 5% carbon dioxide at 37°C. The optimal concentration or half-maximal inhibitory concentration (IC50) values were calculated for all drugs by the MTT assay (8). Finally, the HL-60 cell line was incubated for 48 hours with an optimal concentration of signaling inhibitors.

### 3.3. RNA Isolation and Real-time Polymerase Chain Reaction

Total RNA was extracted using the Denazist Asia kit (Mashhad, Iran) according to the manufacturer’s protocol. Reverse transcription for synthesizing complementary DNA (cDNA) from total RNA was conducted by the Yekta-Tajhiz cDNA synthesis kit (Tehran, Iran) using Molony murine leukemia virus reverse transcriptase and random hexamers. To measure the relative expression level of glucose transporter 1 (GLUT1), hexokinase 2 (HK2), pyruvate kinase M2 (PKM2), pyruvate dehydrogenase E1 (PDHA1), citrate synthase (CS), isocitrate dehydrogenase 2 (IDH2), and hypoxia inducible factor 1 subunit alpha (HIF1A), real-time polymerase chain reaction (PCR) was performed by the amplicon (Copenhagen, Denmark) SYBER Green PCR master mix reagents on a StepOne real-time PCR system (Applied Biosystems, Foster City, CA, USA). The primers were designed by AllelID software and obtained from Metabion International AG (Planegg, Germany) (Table 1). Preliminary experiments were performed to confirm primer efficiencies during reactions, followed by drawing standard curves. Finally, β-actin was used as a housekeeping gene to normalize the data, and the 2^−ΔΔCt^ method was used to calculate the relative expression. All experiments were done in triplicate.

**Table 1.** Primer Sequences in Real-time Polymerase Chain Reaction

Gene	Directions and Sequence (5’-3’)	Product Size (bp)
**GLUT1**	F: GGGTTGTGCCATACTCAT	151 bp
	R: GAAGAGTTCAGCCACGAT	
**HK2**	F: CGGATGTGTATCAATATGGAGT	126 bp
	R: CTTCTCAAACAGTTGCTTTCC	
**PKM2**	F: GGTGTTTGCGTCATTCATC	141 bp
	R: GCCTCCAGGATTTCATCAA	
**PDHA1**	F: AGAGAGGCGATTTCATTCC	180 bp
	R: GTGTACGGTAACTGACTCC	
**CS**	F: GGCTGACCTGATACCTAAG	133 bp
	R: TTCATAGACCAATCCCTTCATG	
**IDH2**	F: CAGGTCCTCAAGTCTTCG	148 bp
	R: CAGCCTCAATCGTCTTCC	
**HIF1A**	F: AGGACAGTACAGGATGCT	136 bp
	R: CTGAATAATACCACTCACAACG	
**β-actin**	F: CCTTCCTGGGCATGGAGTCCT	174 bp
	R: TGGGTGCCAGGGCAGTGAT	

Abbreviations: GLUT1, glucose transporter 1; HK2, hexokinase 2; PKM, pyruvate kinase; PDHA1, pyruvate dehydrogenase E1; CS, citrate synthase; IDH2, isocitrate dehydrogenase 2; and HIF1A, hypoxia inducible factor 1 subunit alpha.

### 3.4. Statistical Analysis

The results were analyzed by GraphPad Prism 9 software. All data were reported as the mean ± standard deviation (SD). Analysis was performed using the Kolmogorov-Smirnov test to determine the normal distribution of the data. A one-way Analysis of Variance (ANOVA) was used, followed by the Bonferroni test for multiple comparisons between the treatment groups and the control group. Pearson’s rank correlation analysis was used to calculate the correlation coefficients. P-values < 0.05 showed statistically significant differences.

## 4. Results

### 4.1. Determination of Optimal Concentration and Time for Treatment of Cells

Following 48 hours of a single treatment of HL-60 cells with different Idelalisib, MK-2206, and Everolimus concentrations, a reduction in viability was observed, and IC50 values were 5 µM, 5 µM, and 2 µM, respectively (8).

### 4.2. Expression of Genes Required for Glucose Uptake and Metabolism Decreased by PI3K/AKT/mTOR Signaling Inhibitors

**Figure 1.**
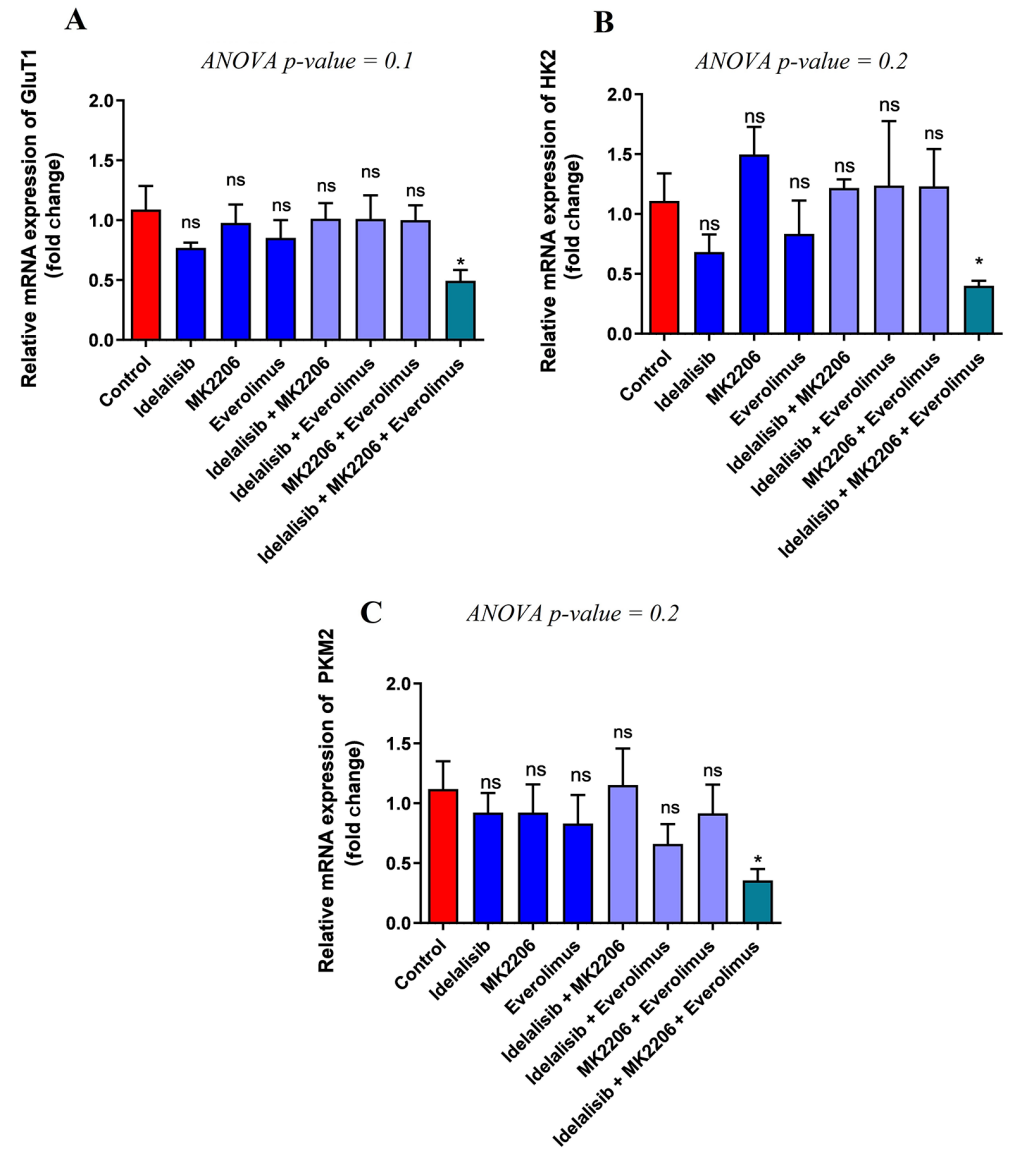
Effect of PI3K/AKT/mTOR pathway inhibitors on the expression of GLUT1, HK2, and PKM2. Expression of GLUT1, HK2, and PKM2 mRNA transcripts in treated HL-60 cells with PI3K/AKT/mTOR signaling inhibitors for 48 hours, either in single or combination, was detected by real-time PCR. To analyze the mean differences between groups, one-way ANOVA was used, followed by a Bonferroni post hoc test to determine differences between the treatment groups and the control group. The results are shown as mean ± SD. ns: Not significant, *P < 0.05.

The effects of PI3K, AKT, and mTOR inhibitors on the transcription levels of genes involved in glucose transport and glycolysis in cancerous cells, including GLUT1, HK, and PKM2, were evaluated. The results only showed a significant reduction in GLUT1 after exposure of HL-60 cells to the combination of Idelalisib, MK-2206, and Everolimus compared to the control group. However, no significant difference was obtained comparing the expression of GLUT1 after the treatment individually or in combination with two signaling pathway inhibitors (Figure 1A). Similar to GLUT1, the results demonstrated a significant downregulation of HK2 in combined treatment with Idelalisib, MK-2206, and Everolimus, but a considerable difference was not observed among other groups (Figure 1B). Subsequently, the expression of PKM2 also indicated a significant decrease after treatment with the combination of three signaling pathway inhibitors. However, similar to GLUT1 and HK2, no significant difference in the expression level of PKM2 was observed among the remaining groups (Figure 1C). Finally, considering the above results, using Idelalisib, MK-2206, and Everolimus simultaneously showed a significant decrease in glucose transport and metabolism.

### 4.3. PI3K/AKT/mTOR Inhibitors Alter Gene Expression in the Pyruvate Dehydrogenase Complex and TCA Cycle

**Figure 2.**
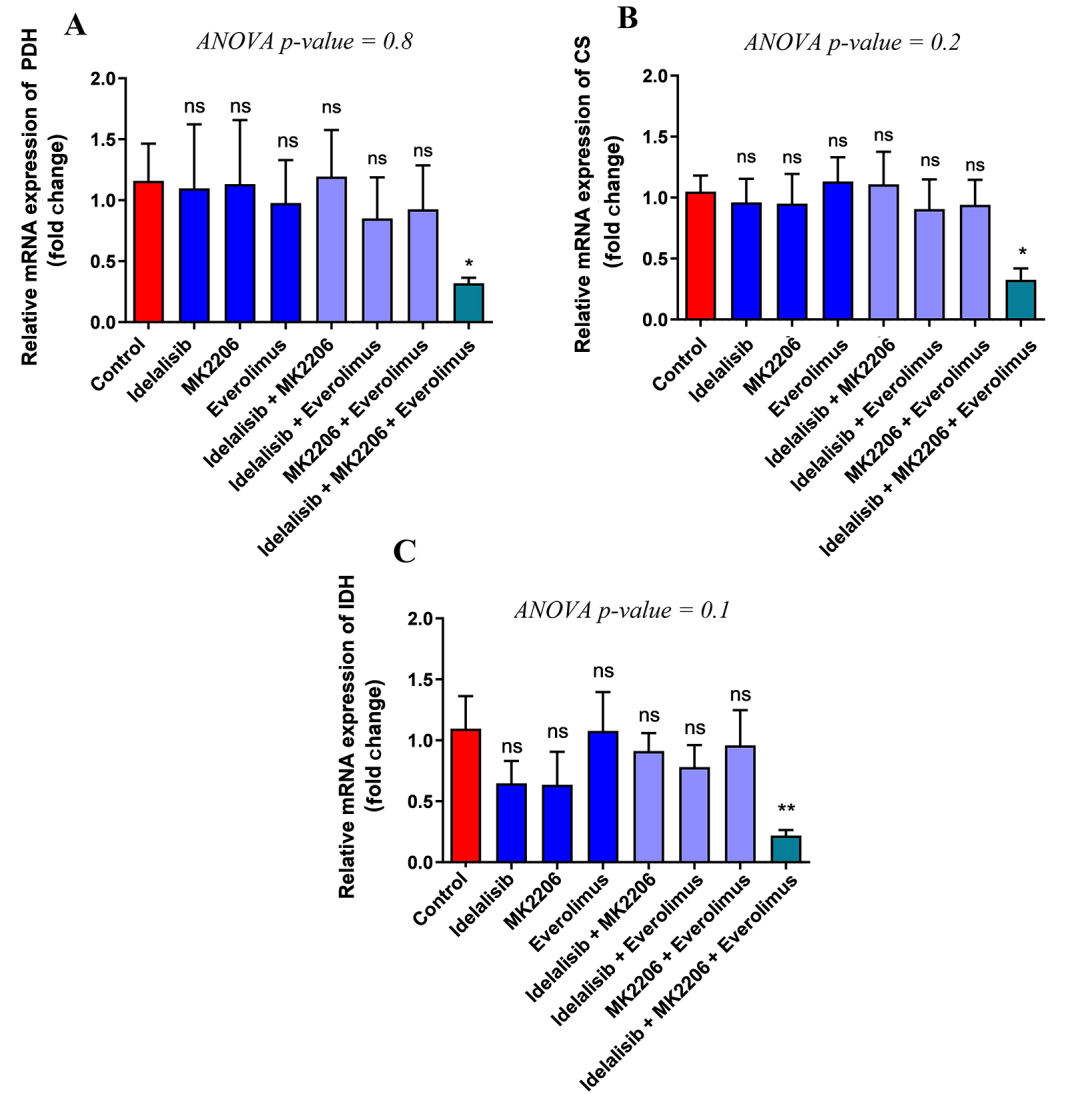
Effect of PI3K/AKT/mTOR pathway inhibitors on the expression of PDH, CS, and IDH. Expression of PDH, CS, and IDH mRNA transcripts in treated HL-60 cells with PI3K/AKT/mTOR signaling inhibitors for 48 hours, either in single or combination, was detected by real-time PCR. To analyze the mean differences between groups, one-way ANOVA was used, followed by a Bonferroni post hoc test to determine differences between the treatment groups and the control group. The results are shown as mean ± SD. ns: Not significant, *P < 0.05.

For further investigation of the impact of PI3K, AKT, and mTOR signaling pathway inhibitors after the metabolism of glucose in the glycolysis pathway and entering the pyruvate dehydrogenase complex (PDH) and TCA cycle, the expression levels of the following genes were studied: Pyruvate dehydrogenase E1 (PDHA1), citrate synthase (CS), and isocitrate dehydrogenase 2 (IDH2). The results showed a significant decrease in PDHA1 as part of the PDH complex and the relative expression following treatment with Idelalisib, MK-2206, and Everolimus together compared to the control group, but no significant difference was indicated between other groups (Figure 2A). Also, the results showed a significant decrease in the expression level of CS as part of the TCA cycle in combination with the three above drugs. However, no considerable difference in groups, whether single or co-treated with two drugs, was indicated (Figure 2B). The results of IDH, another gene involved in the TCA cycle, demonstrated an expression reduction after exposure to signaling inhibitors at the same time. Nevertheless, similar to CS, there was no decrease in IDH expression in other groups (Figure 2C). Regarding the results, only a combination of PI3K/AKT/mTOR inhibitors could decrease the genes involved in the PDH complex and TCA cycle.

### 4.4. Effect of PI3K/AKT/mTOR Inhibitors on HIF-1α Expression

**Figure 3.**
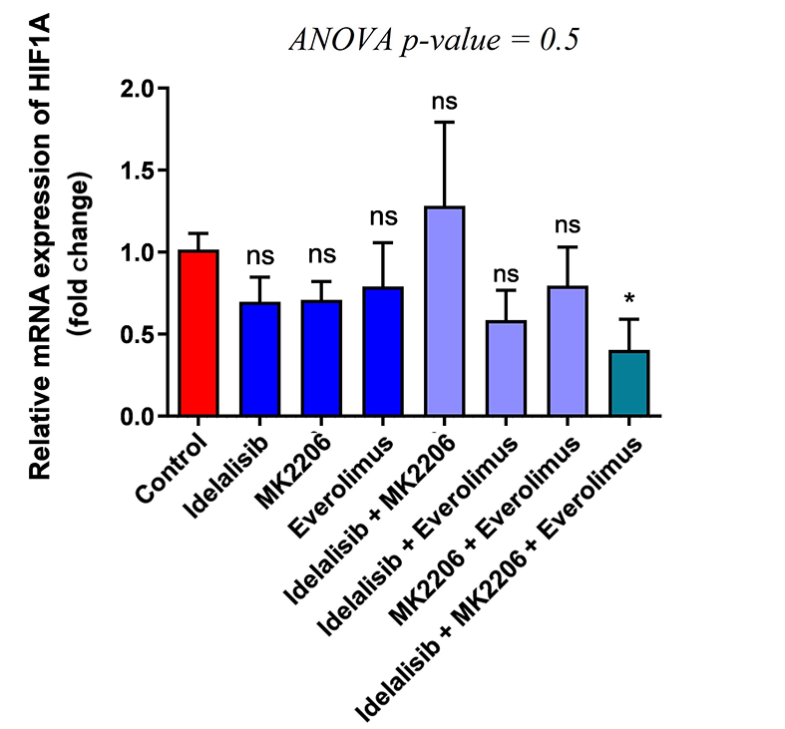
Effect of PI3K/AKT/mTOR pathway inhibitors on the expression of HIF1-α. Expression of HIF1-α mRNA transcripts in treated HL-60 cells with PI3K/AKT/mTOR signaling inhibitors for 48 hours, either in single or combination, was detected by real-time PCR. To analyze the mean differences between groups, one-way ANOVA was used, followed by a Bonferroni post hoc test, to determine differences between the treatment groups and the control group. The results are shown as mean ± SD. ns: not significant, *P < 0.05.

As HIF1-α plays a vital role in the transcription of different genes involved in glucose and energy metabolism, the expression level of HIF-1α was evaluated to reveal the impact of Idelalisib, MK-2206, and Everolimus. The results showed a significant decrease while HL-60 cells were treated using the three above drugs. However, similar to other results obtained before, no difference was seen in other groups (Figure 3). Furthermore, regarding these results, inhibiting the whole PI3K, AKT, and mTOR signaling pathways together demonstrated considerable downregulation of HIF1-α expression, but not individually. Finally, the results showed a positive association between the reduction of glucose uptake and energy metabolism through glycolysis, the PDH complex, and the TCA cycle when applying the PI3K, AKT, and mTOR signaling pathway inhibitors.

### 4.5. Expression of Immune Checkpoint Ligands Was Positively Correlated with the Expression of Metabolic Genes in HL-60 Treated with Combined PI3K/AKT/mTOR Inhibitors

**Figure 4.**
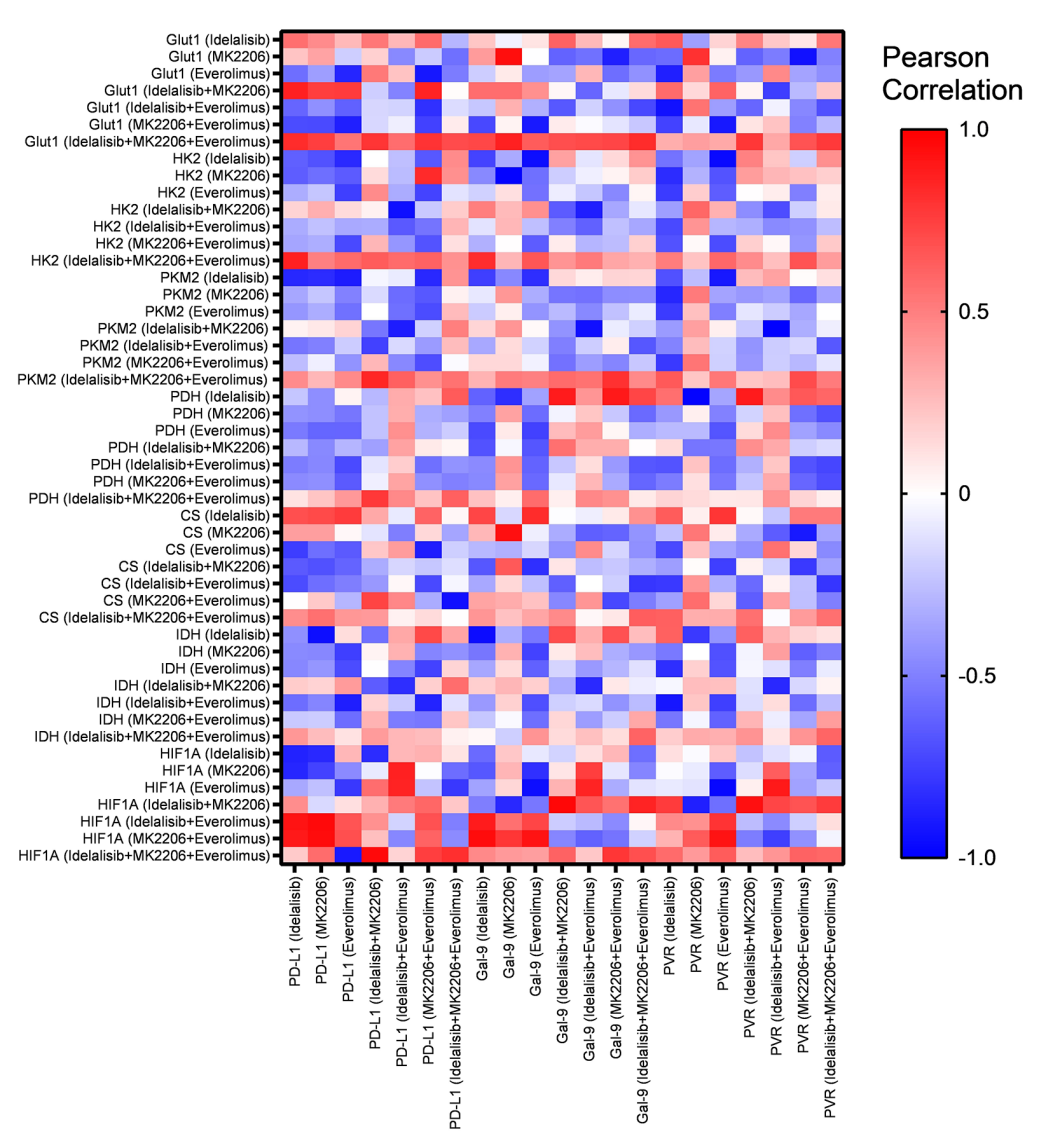
Heat map of correlation analysis based on transcriptome data of immune checkpoint ligands and glucose metabolic genes following treatment with PI3K/AKT/mTOR pathway inhibitors.

In our previous study, we showed that the expression of PD-L1, Gal-9, and CD155 as immune checkpoint ligands was decreased in HL-60 cells after treatment with Idelalisib, MK-2206, and Everolimus alone or in combination when compared to the untreated group (8). The current results were analyzed with our previous data to find any correlations between the expression of immune checkpoint ligands and the expression of glucose metabolic genes, including GLUT1, HK2, PKM2, PDH, CS, IDH, and HIF1-α. After combined treatment of HL-60 with three PI3K, AKT, and mTOR inhibitors, PD-L1, Gal-9, and CD155 expressions were positively correlated with GLUT1, HK2, PKM2, and HIF1-α (Figure 4). The heat map shown in Figure 4 displays the correlations in more detail.

## 5. Discussion

Alterations in metabolic pathways and taking advantage of them for growth, survival, and proliferation in the tumor microenvironment are prominent features of cancer cells (14). Reprogramming metabolism toward the high proliferation rate in cancer cells is essential for supplying energy and the building blocks to synthesize nucleic acids, lipids, and proteins (14). Also, changing metabolic patterns involves reducing the required nutrients and producing metabolites that impair the proper functioning of immune cells, causing defects and suppressing the immune response against the tumor (15). Elucidation of the metabolic properties of cancerous cells and the underlying mechanisms can be used to design new treatment strategies and overcome current drug failures (15). Active intracellular signaling pathways, regulated by mutant oncogenes and genes suppressing tumors, cause a metabolic rewiring in cancerous cells (16). Considering the advantages of targeted therapies over traditional therapies, inhibition of these signaling pathways, which play an essential role in tumor cell activity, by small-molecule inhibitors has become part of standard treatment (17). However, the role of these inhibitors in metabolic pathways has not been fully understood. Accordingly, our data demonstrated that a combination of PI3K/AKT/mTOR pathway inhibitors regulates the expression of genes involved in glycolysis, the PDH complex, and the TCA cycle and interferes with metabolic reprogramming of AML leukemic cells.

Many cancer cells use glycolysis under normoxic and hypoxic conditions to provide energy, which today is called aerobic glycolysis (18). Accordingly, other studies demonstrated the upregulation of related genes in glycolysis as a resistance mechanism to chemotherapy drugs in AML patients and the AML cell line (19, 20). On the other hand, targeting the PI3K/AKT/mTOR pathway may have anti-apoptotic and anti-coagulant effects in hematological malignancies, although the best strategy to inhibit this pathway remains to be elucidated (21). However, one of the possible mechanisms is related to the increase in glycolysis (21). Accordingly, the PI3K/AKT/mTOR signaling pathway was recently identified to considerably regulate glucose uptake and metabolism through glycolysis in cancerous cells (7). Our result showed that the combined use of PI3K, AKT, and mTOR inhibitors resulted in a significant reduction in the expression of the genes GLUT1, HK2, and PKM2. Moreover, previous studies showed the significant role of the PI3K/AKT/mTOR pathway in the regulation and expression of GLUT1, HK2, and PKM2, leading to the downregulation of glycolytic activity in AML cell lines (13).

About a century ago, Warburg showed that tumor cells preferred to use glycolysis and convert glucose to lactate even in the presence of sufficient oxygen due to the existence of defects in mitochondria. It is now widely established that reprogramming of the metabolic pathways occurring in mitochondria rather than simple dysfunction is essential for tumor growth (13). Further studies showed that to meet the anabolic metabolites, cancer cells enter 10 - 15% glucose carbons into the TCA cycle (22). Furthermore, the PDH complex plays a pivotal role in supplying carbon into the TCA cycle by converting glucose-derived pyruvate into acetyl-CoA, and inhibiting the PDH complex is a therapeutic strategy (23). The PI3K/AKT/mTOR signaling pathway reprograms the metabolism of mitochondria in AML (24). In this line, our results demonstrated that inhibiting PI3K/AKT/mTOR simultaneously resulted in a significant reduction in CS, IDH2, and PDHA1. Consistent with our result, another study showed that inhibition of PI3K/mTOR or genetic inhibition of AKT/PI3K increased the phosphorylation of PDHE1 and resulted in the inhibition of PDH complex activity (25).

On the other hand, it has long been known that signaling pathways and transcription factors play an essential role in metabolic alteration in cancerous cells, and AML is no exception in this case. HIF-1α is one of the critical transcription factors involved in metabolic reprogramming through glucose transport, glycolysis, the PDH complex, and the TCA cycle in tumor cells (26). As a result, it can be suggested that HIF-1α promotes the Warburg effect in cancer cells (27). Moreover, different studies showed the PI3K/AKT/mTOR pathway's role in the activation of HIF-1α, although HIF-1α expression could be regulated by other pathways (28). Consistently, our results showed a considerable reduction in HIF-1α expression during the exposure to PI3K/AKT/mTOR inhibitors together. This reduction was consistent with the reduced expression of GLUT1, HK2, PKM2, PDHA1, CS, and IDH2.

Some studies have shown that, in immune and tumor cells, immune checkpoints act in part by metabolic reprogramming (29). Our previous studies revealed that PI3K/AKT/mTOR inhibitors are not only cytotoxic agents but also control the immune checkpoint ligand expression and disrupt the immune escape mechanisms of AML cells (8). Accordingly, our correlation analysis of gene expression demonstrated that several metabolic genes, including GLUT1, HK2, PKM2, and HIF-1α, were significantly and positively correlated with the expression of immune checkpoint ligands such as PD-L1, Galectin-9, and CD155 in HL-60 cells after combinational treatment with PI3K/AKT/mTOR inhibitors. Interestingly, recent data show that hypoxia can upregulate the expression of PD-L1 in tumor cells via HIF-1α and PKM2 (30, 31). Therefore, these findings revealed that the interaction between immune checkpoints and their ligands also regulates the metabolism of the immune and tumor cells.

Metabolic reprogramming is recognized as a hallmark of cancer and allows cancer cells to survive in the tumor microenvironment while fulfilling their anabolic requirements. On the other hand, the over-activation of PI3K/AKT/mTOR signaling pathways in tumor cells is associated with metabolic rewiring and immune evasion mechanisms. Based on the results obtained from this study, it can be concluded that interfering with the metabolic changes of cancerous cells with combination therapy to block the PI3K/AKT/mTOR signaling pathway could be a novel therapeutic strategy.

## Data Availability

The dataset presented in the study is available on request from the corresponding author during submission or after publication. The data are not publicly available due to privacy and ethics.
